# The Role of the Gastrointestinal Mucus System in Intestinal Homeostasis: Implications for Neurological Disorders

**DOI:** 10.3389/fcimb.2020.00248

**Published:** 2020-05-28

**Authors:** Madushani Herath, Suzanne Hosie, Joel C. Bornstein, Ashley E. Franks, Elisa L. Hill-Yardin

**Affiliations:** ^1^Department of Physiology, University of Melbourne, Parkville, VIC, Australia; ^2^School of Health and Biomedical Sciences, RMIT University, Bundoora, VIC, Australia; ^3^School of Life Sciences, La Trobe University, Bundoora, VIC, Australia

**Keywords:** mucus, MUC-2, goblet cells, intestine, microbes, neurological disorders

## Abstract

Mucus is integral to gut health and its properties may be affected in neurological disease. Mucus comprises a hydrated network of polymers including glycosylated mucin proteins. We propose that factors that influence the nervous system may also affect the volume, viscosity, porosity of mucus composition and subsequently, gastrointestinal (GI) microbial populations. The gut has its own intrinsic neuronal network, the enteric nervous system, which extends the length of the GI tract and innervates the mucosal epithelium. The ENS regulates gut function including mucus secretion and renewal. Both dysbiosis and gut dysfunction are commonly reported in several neurological disorders such as Parkinson's and Alzheimer's disease as well in patients with neurodevelopmental disorders including autism. Since some microbes use mucus as a prominent energy source, changes in mucus properties could alter, and even exacerbate, dysbiosis-related gut symptoms in neurological disorders. This review summarizes existing knowledge of the structure and function of the mucus of the GI tract and highlights areas to be addressed in future research to better understand how intestinal homeostasis is impacted in neurological disorders.

## Properties of the Gastrointestinal Mucus Layer

The mucus layer is the first line of defense against infiltration of microorganisms, digestive enzymes and acids, digested food particles, microbial by-products, and food-associated toxins. This layer coats the interior surface of the GI tract, lubricates luminal contents and acts as a physical barrier to bacteria and other antigenic substances present in the lumen. The moist, nutrient-rich mucus layer adjacent to the epithelial barrier of the GI tract is also essential in the maintenance of intestinal homeostasis and contains a thriving biofilm including beneficial and pathogenic microbial populations.

Emerging evidence demonstrates changes in the gut-brain axis in neurological disease involving the enteric nervous system located within the wall of the GI tract. Interestingly, mucus production is regulated by molecular pathways involved in developmental processes and nervous system activity. Multiple neurological disorders present with gastrointestinal dysfunction and microbial dysbiosis but whether alterations in mucus structure and function are driving these changes is unknown. Therefore, we propose that alterations in enteric nervous system function and mucus production may occur in neurological disease and contribute to GI symptoms and dysbiosis.

### Regional Mucus Variations

Although mucus located throughout the gut contains the same biological components, mucus properties vary with regional differences in function along the gastrointestinal tract (Ermund et al., 2013, Figure 1).

**Figure 1:**
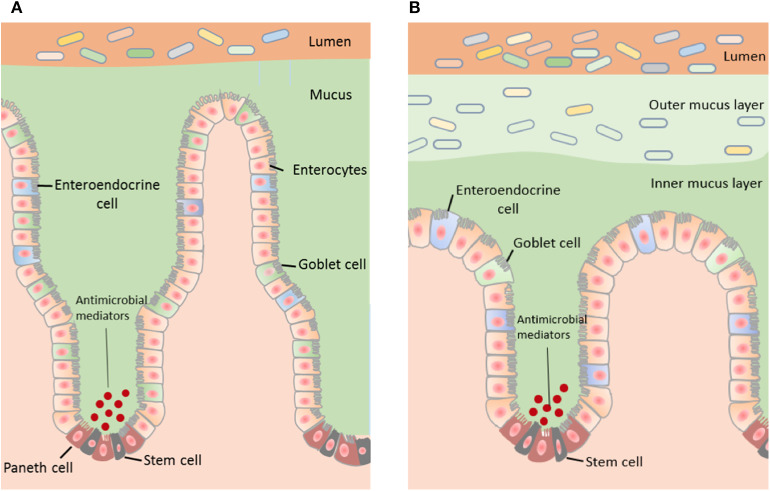
The structure of the mucus layer varies with regional locations within the GI tract. **(A)** The small intestine contains a single layer of mucus, which is loosely attached to the epithelium and easily penetrable. Bacteria within the small intestine are primarily repelled from the epithelium by antibacterial modulators. **(B)** The distal colon contains two mucus layers; a stratified adherent inner mucus layer and loosely adhesive outer mucus layer. The inner mucus layer of the colon is essentially sterile and the outer mucus layer harbors the intestinal microbiota.

#### Small Intestine

The majority of nutrient uptake from digested food occurs in the small intestine and therefore there is a single, discontinuous and more penetrable mucus layer in this region (Johansson et al., 2011). The discontinuity of the small intestinal mucus layer is important not only for the absorptive function of this region but also for the release of digestive enzymes localized in the brush border membrane of epithelial cells. Experiments assessing passage of fluorescent beads across small intestinal mucosal samples showed that small intestinal mucus in mice is penetrable by beads equivalent to the size of bacteria (i.e., 0.5–2 μ^3^) and hence contains pores as large as 2 μ^2^ (Ermund et al., 2013). These large mucus pores ensure efficient nutrient absorption by the host epithelium.

The bacterial content of the mucosal barrier in the small intestine is also regulated by a cocktail of antibacterial mediators such as defensins, lysozymes, and other peptides released by Paneth cells (Peterson et al., 2007). Together, these mediators repel bacteria by generating an antibacterial gradient toward the lumen (Johansson and Hansson, 2011; Vaishnava et al., 2011). Specific mediators include the abundant Regenerating islet-derived 3 (REG3) peptides, IgA, Toll-like receptor 5 (TLR5 regulates levels of anti-flagellin antibody in the gut) (Cullender et al., 2013) and phospholipase A2-IIA (Meyer-Hoffert et al., 2008; Bevins and Salzman, 2011). Overall, antibacterial peptides kill bacteria via a range of mechanisms including by the formation of aggregates, recognition, and binding to bacterial cell wall peptidoglycans, and permeabilization of bacterial cell membranes (Chairatana and Nolan, 2017). This serves to neutralize invasion by foreign particles and maintain epithelial crypts. This antimicrobial defense mechanism is critical in the small intestine due to the discontinuous and penetrable nature of the mucus in this region and is reflected by a higher density of Paneth cells and corresponding peptides (Ouellette, 2010).

#### Colon

The organization of the mucus layer varies along the length of the colon. In the distal colon, there are two layers of mucus, however, whether these layers adhere to the epithelium or the colonic content is under debate. In the proximal colon, the presence of two mucus layers has been queried based on histological studies in animal models.

Johansson and colleagues reported that the mouse distal colon contains two continuous mucus layers; an inner mucus layer that is ~50 μm thick and anchored to the mucus-producing goblet cells of the epithelial membrane, and an outer mucus layer that is loosely adherent and harbors bacteria (Johansson et al., 2008). These researchers also reported that the thickness of the outer mucus layer is determined by the composition of the mucus-inhabiting bacteria. Interestingly, this group reported that the inner mucus layer of the proximal colon is also penetrable to bacteria (Ermund et al., 2013). In contrast, Kamphuis and colleagues reported that the two distal colonic mucus layers adhere to the fecal pellet rather than the intestinal epithelium in rodents and that the organization of the colonic mucus layers is dependent on the presence of fecal content (Kamphuis et al., 2017). Specifically, this study utilized fluorescence *in situ* hybridization and histological techniques in longitudinal sections to demonstrate that the fecal pellet is covered by a sterile mucus layer of variable thickness that is not attached to the epithelium. They also showed that within the proximal part of the proximal colon, which contains colon content prior to formation of a fecal pellet, the mucus layer is loosely organized and the bacteria in this region are in contact with the epithelial surface (Kamphuis et al., 2017).

The dissimilarities in the mucus layers of the colon reported may be due to methodological variations including the orientation of tissue sectioning and mucus staining techniques. Overall, multiple studies examining mucus properties carried out in both mice (Macfarlane et al., 2011; Motta et al., 2015; Welch et al., 2017) and humans (Swidsinski et al., 2007a) describe two mucus layers in the colon that include a firm mucus layer adjacent to the epithelium that is devoid of bacteria.

Commensal bacteria secrete mucinases and proteinases that continuously degrade the outer mucus layer contributing to its highly disorganized nature (Donaldson et al., 2016). Similarly, a role for bacteria in mucus thickness has been demonstrated in germ free mice which have a thinner inner colonic mucus layer. Simply adding components of the bacterial cell wall (e.g., lipopolysaccharide; LPS) is sufficient to increase mucus thickness in this model, highlighting a role for bacteria in regulating the structure of the outer mucus layer (Petersson et al., 2011). The continual release of mucus contributes to a dynamic process whereby the inner mucus layer is gradually converted to the irregular and less adherent outer mucus layer. This process involves Meprin β, an endogenous protease which aids mucus detachment (Wichert et al., 2017) and also bacteria penetration by increasing pore size in the outer mucus layer (Schutte et al., 2014).

### Intestinal Mucus Composition

Mucus is primarily composed of branched glycoproteins (including mucins) that interact with the external environment and via their hydrophilic nature, influence mucus viscosity (Bergstrom and Xia, 2013). There are more than 20 subtypes of mucin identified in humans and their distribution varies throughout the GI tract. For example, the salivary glands produce MUC5B and MUC7 to lubricate food (Bobek et al., 1993; Nielsen et al., 1996; Khan et al., 1998; Thornton et al., 1999) and the mucus layer in the stomach contains MUC5AC (Ho et al., 1995; Atuma et al., 2001; Nordman et al., 2002). Although MUC5AC is not typically expressed in the large intestine, it has been detected in the distal colon along with MUC-2 during inflammation associated with ulcerative colitis and adenocarcinoma in patients (Forgue-Lafitte et al., 2007). It is well-established that the major glycoprotein within the intestinal mucus layer is mucin-2 (MUC-2 protein).

**Figure 2:**
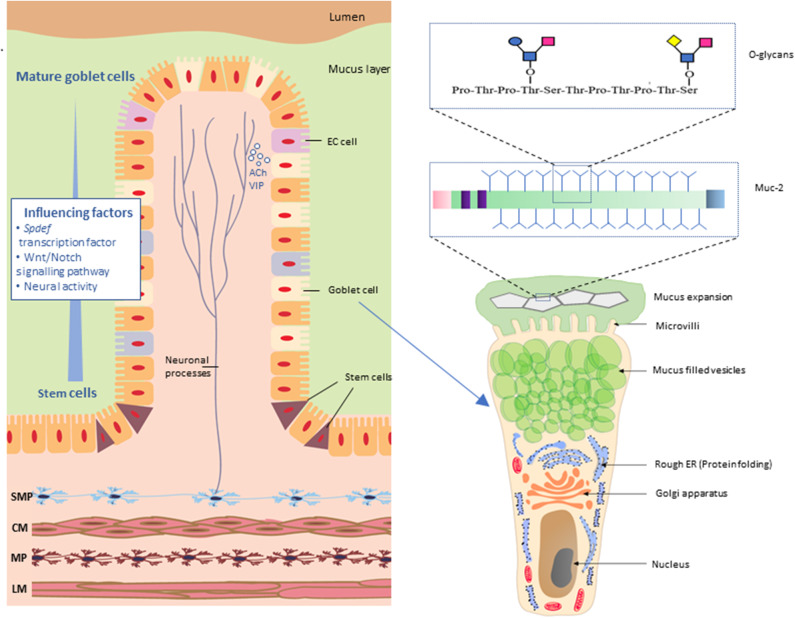
Neuronal innervation of goblet cells in the intestinal mucosa. Neurons of the submucosal plexus (SMP) innervate goblet cells by release of neurotransmitters such as acetylcholine (ACh) and vasoactive internal peptide (VIP). Maturation of goblet cells is influenced by *SAM pointed domain-containing Ets transcription factor* (*Spdef*), Wnt/Notch signaling and neuronal activity. Mature goblet cells have a characteristic goblet shape. The apical region is distended by the presence of mucin granules, giving the cell the characteristic cup shape with other cellular organelles condensed in the basal “stem-like” region. Muc-2 protein comprises multiple O-glycans arranged in a “bottle brush” like formation. SMP, submucosal plexus; CM, circular muscle; MP, myenteric plexus; LM, longitudinal muscle; EC cell, enteroendocrine cells.

There are three major structural domains within the MUC2 protein; the N-terminal domain, a central large PTS (proline, threonine, and serine) domain and the C-terminal domain. Following translation, full-length MUC2 protein cores form dimers via disulfide bridges near their C-terminus within the endoplasmic reticulum (ER) of goblet cells. Within the Golgi apparatus, MUC2 proteins undergo O-linked glycosylation. In this process glycans such as xylose, mannose, N-acetylglucosamine, and N-acetylgalactosamine (O-GalNAc) are covalently attached to the hydroxyl group (-OH) of threonine and serine residues of the PTS domain (Godl et al., 2002). Glycans account for 80% of the total mass of the MUC2 protein and extend perpendicularly from the protein core giving the molecule a “bottle brush-like” appearance (Figure 2). O-Glycans can be modified via formation of linkages with sulfate, sialic acid, and fucose. These modifications play an important role in influencing interactions between the host microbial populations with mucus (Arike and Hansson, 2016).

A complex polymerization process occurs within the trans-Golgi network by which MUC2 protein dimers interact firstly as trimers and then are tightly bundled into MUC2 secretory granules (Godl et al., 2002; Ambort et al., 2012). High Ca^2+^ ion concentration alongside low pH enables mucus packing by masking negatively charged glycans on the MUC2 protein. During this process, concatenated ring structures are formed (Grubb and Gabriel, 1997; Choi et al., 2001; Ambort et al., 2012; Gustafsson et al., 2012b; Schutte et al., 2014).

Although the main component of mucus in the small intestine and the colon is mucin-2, a rich variety of other proteins largely originating from shredded epithelial cell debris that becomes trapped in the mucus are also present within the mucus biofilm, including IgG Fc-binding protein (FCGBP), Calcium activated chloride channel 1 (ClCA1), Zymogen granule membrane protein 16 (ZG16), Anterior gradient 2 (AGR2), and immunoglobulins (Johansson et al., 2008).

#### Mucus Expansion

After mucus secretion, the MUC2 protein complex expands dramatically to form a net-like structure (Ambort et al., 2012). Mucin expansion occurs due to increased pH and decreased Ca^2+^ levels driven by cystic fibrosis transmembrane regulator (CFTR) channels. CFTR-mediated secretion of HCO3^−^ reduces Ca^2+^ levels which weakens the ring structure of the mucin complex and allows the densely packed MUC2 mucin to expand into large flat sheets (Ambort et al., 2012). The newly secreted mucus sheets are laid down on the epithelium by interacting with previously secreted mucus and subsequently attaching to the epithelium (Johansson and Hansson, 2016) (Figure 2). In the colon, expansion of the outer mucus layer is also triggered by bacteria that release glycosidases that sequentially cleave individual monosaccharides from mucin glycans (Johansson and Hansson, 2016) to further relax the tight-knit structure of mucin glycans (Johansson et al., 2008).

#### Mucus Secreting Goblet Cells

The intestinal epithelium consists of absorptive and secretory cell lineages including enterocytes, enteroendocrine cells (EECs), Paneth cells, and goblet cells. Goblet cells are specialized cells equipped with specific biological machinery for the secretion of mucus and are present throughout the entire length of the intestine (Figure 2). These cells, as their name suggests, are easily identifiable in histologically stained cross sections of the intestine due to their characteristic “goblet-like” shape. Intestinal epithelial cells, including goblet cells, arise from multipotential stem cells residing at the base of the intestinal crypts and subsequently migrate from the crypts to the top of the villus prior to eventually being shed into the lumen (Cheng and Leblond, 1974). In mice, this migratory process occurs over 2–3 days (Specian and Oliver, 1991). Differentiation of goblet cells is directly controlled by the transcription factor *SAM pointed domain-containing ETS transcription factor* (*Spdef*) (Noah et al., 2010) and also via a network of transcriptional factors regulated by the Notch and Wnt signaling pathways known to influence developmental and inflammation pathways (van Es et al., 2005; Clarke, 2006; Fre et al., 2009; Gersemann et al., 2009; Gregorieff et al., 2009; Kwon et al., 2011; Heuberger et al., 2014; Tian et al., 2015). Furthermore, enteric neural activity has been shown to influence the maturation and production of stem cells in the GI tract (Lundgren et al., 2011) which, in turn, suggests a role for the ENS in goblet cell proliferation and differentiation.

Goblet cell morphology changes dramatically during the cellular lifespan (Specian and Oliver, 1991). Immature goblet cells are larger and pyramidal in shape with cellular organelles dispersed throughout the cell and interspersed with mucus granules in the apical cellular region. As these goblet cells migrate toward the colonic epithelial surface, they reduce in volume as a result of shedding cytoplasmic content and organelles. During this phase of volume reduction, goblet cells reduce contact with the basal laminar surface adjacent to the epithelium and simultaneously increase contact with the luminal surface of the GI tract. The goblet cells then rapidly produce and store mucus granules, resulting in the distention of the apical cellular region to produce the typical “cup” shape. The nucleus and other cellular organelles of the goblet cells are concentrated in narrowed stem-like subcellular regions located at the base of the cells (Specian and Oliver, 1991). These processes could be altered in neurological disorders. For example in Alzheimer's disease, the metalloprotease Meprin β, which cleaves amyloid precursor protein (Schönherr et al., 2016; Becker-Pauly and Pietrzik, 2017) also regulates mucus detachment from goblet cells in the small intestine (Wichert et al., 2017).

#### Mucus Interactions With Microbes

Microbial populations are spatially organized along the length of the intestine as well as from the luminal to mucosal axis (Palestrant et al., 2004). Mucus viscosity increases toward the distal region of the GI tract. This viscosity gradient along the length of the GI tract reportedly determines the spatial distribution of intestinal microbiota (Swidsinski et al., 2007b). The composition of bacteria adjacent to the mucosa is different to the bacterial populations that reside within the luminal content (Swidsinski et al., 2005). This mucosal to luminal bacterial distribution is likely driven by variations in oxygen levels and nutrient availability (Yasuda et al., 2015).

The mucus layer serves as a carbon and energy source, predominantly in the form of glycans, for mucus residing bacteria. As an adaptation to residing in a glycan-rich environment, these bacteria produce mucus-degrading enzymes such as glycosidase, sulphatase, and sialidases (Table 1) that cleave the mucus network to enhance the utilization of mucus as an energy source. A range of mucus-degrading bacteria present within the mucus, includes *Akkermansia muciniphila* (Derrien et al., 2004), *Bacteroides thetaiotaomicron* (Xu et al., 2003), *Bifidobacterium bifidium* (He et al., 2001), *Bacteroides fragilis* (Macfarlane and Gibson, 1991), and *Ruminoccous gnavus* (Png et al., 2010). These bacterial species cleave mucus O-glycans to produce monosaccharides (Berry et al., 2013) which can be further utilized by other mucus-residing bacteria including Lachnospiraceae (Nava et al., 2011), Clostridium cluster XIV (van den Abbeele et al., 2013), Enterobacteriaceae (Ashida et al., 2008), and *Clostridium difficile* (Ng et al., 2013). Further adaptation of bacteria has been identified in Lactobacillus (Etzold et al., 2014) and Bacteroides (Sicard et al., 2017) where the presence of multi-repeat cell-surface adhesins enable retention of the bacteria within the mucus layer. The syntrophic, symbiotic, and mutualistic interactions of the microbes in the mucus layer create the environment which drives microbial community selection and defines physical properties of the mucus layer.

**Table 1:** Predominant mucus-degrading bacteria and secreted digestive enzymes.

**Bacteria**	**Mucus degrading enzyme**	**References**
*Akkermansia muciniphila*	Glycosidase	Png et al., 2010; van Passel et al., 2011
*Bacteroides thetaiotaomicron*	Sulfatase, neuraminidase, α-fucosidase, β-galactosidase α- N-acetylgalactosaminidase β-N-acetylglucosaminidase	Xu et al., 2003
*Rumminococcus gnavus*	α-galactosidases	Png et al., 2010
*Rumminococcus torques*	α-N-acetylgalactosaminidase	Png et al., 2010
*Bacteroides fragilis*	Neuraminidase, sulfatase, protease, α- N-acetylgalactosaminidase, β-galactosidase, β -N-acetylglucosaminidase, α-fucosidases	Macfarlane and Gibson, 1991
*Bacteroides vulgatus*	Neuraminidase, α and β-galactosidases, α-fucosidase β-N-acetylglucosaminidase, α and β -N-acetylgalactosaminidase	Onderdonk et al., 1983; McCarthy et al., 1988
Adherent invasive *Escherichia coli*	Vat protease	Gibold et al., 2016
*Giardia duodenalis*	Cysteine protease	Amat et al., 2017
*Entamoeba histolytica*	Cysteine protease	Lidell et al., 2006

Some mucus residing bacteria form mucosal biofilms, complex microbial communities embedded in a polymeric matrix. Techniques including fluorescent *in situ* hybridization and electron microscopic studies reported the presence of bacterial biofilms in the healthy colon of mice, humans and rats (Palestrant et al., 2004; Swidsinski et al., 2005; Bollinger et al., 2007; Macfarlane et al., 2011; Motta et al., 2015). Altered levels of biofilm associated bacteria such as *Bacteroides fragilis*, Enterobacteriaceae family were reported in Crohn's disease and inflammatory bowel disease (Masseret et al., 2001; Macfarlane and Dillon, 2007; DuPont and DuPont, 2011; Srivastava et al., 2017).

Therefore, the mucus associated bacterial biofilm also could play a role in these disorders. Alterations in these complex community structures could result in abnormal mucus invasion, epithelial adherence, and spatial distribution of bacterial species.

## The Enteric Nervous System (ENS)

The digestive tract is innervated by the enteric nervous system (ENS), an intrinsic neuronal network that regulates GI functions (Furness et al., 2013) in addition to extrinsic innervation from the parasympathetic and sympathetic components of the autonomic nervous system (reviewed in Uesaka et al., 2016). Neuronal control of intestinal function is largely regulated by two ganglionated plexuses; the myenteric and submucosal plexus. The myenteric plexus predominantly regulates GI motility while the submucosal plexus regulates the secretion of water and electrolytes primarily via the neurotransmitters acetylcholine (ACh) and vasoactive intestinal peptide (VIP).

### The ENS Influences Mucus Secretion

Mucus secretion is influenced by nervous system activity and occurs via two processes; (i) vesicle secretion and (ii) compound exocytosis. During vesicle secretion, mucus-secreting goblet cells release mucus content by fusion of the mucus granule membrane with the overlying plasma membrane (Lang et al., 2004). This process is regulated by vesicle exocytotic components like syntaxin, Munc 18, vesicle-associated membrane proteins (VAMP) and synaptosomal nerve-associated proteins (SNAP) proteins (Cosen-Binker et al., 2008). During compound exocytosis, all mucus granules are fused together and empty the mucus as a single unit. As yet, the molecular pathways regulating compound exocytosis have not been defined.

VIP and ACh are the two main secretagogues responsible for neurally-evoked mucosal secretion (Specian and Neutra, 1980; Neutra et al., 1984; Lelievre et al., 2007; Gustafsson et al., 2012a; Ermund et al., 2013). ACh induces mucus secretion by activating M3 muscarinic receptors located on goblet cells within the epithelium in both the small intestine and in the colon (Specian and Neutra, 1980; Neutra et al., 1984; Gustafsson et al., 2012b; Ermund et al., 2013). Exocytosis of mucus-containing granules is regulated by intracellular Ca^2+^ and Ca^2+−^mobilizing agents (including acetylcholine; Birchenough et al., 2015). The activation of M3 muscarinic receptors mobilizes Ca^2+^ from intracellular stores to induce mucus secretion (Ambort et al., 2012).

Mucus release is differentially regulated in a region-specific manner in the GI tract. ACh specifically targets both crypt and villus-associated goblet cells in the small intestine (Birchenough et al., 2015). In contrast, in the colon, goblet cells located in crypts are responsive to ACh, but equivalent cells at the epithelial surface do not respond to ACh or the cholinergic agonist, carbachol (Gustafsson et al., 2012b). Release of the neuropeptide VIP enhances mucus secretion (Lelievre et al., 2007) via modulating CFTR-dependent secretions (Alcolado et al., 2014). Furthermore, VIP deficiency in mice results in reduced goblet cell number and reduced *muc-2* gene expression levels (Wu et al., 2015). A recent study displayed that mucosal VIP-containing neurons are in close proximity with ileal goblet cells and VPAC receptor antagonist alter the goblet cell numbers in the ileum (Schwerdtfeger and Tobet, 2020).

### Gut Motility and Mucus Movement

In addition to its prominent action in regulating GI motility and peristalsis, the myenteric plexus plays a key role in mucus renewal. GI motility regulates mucus levels by propelling mucus to the distal GI tract. Myenteric neurons coordinate cyclic motility patterns known as migrating motor complexes (MMCs) that contribute to the “housekeeping” functions of the intestine by flushing undigested materials, mucus, and bacteria along the small intestine. Altered ENS regulation of motility can therefore also perturb mucus renewal. Interestingly, patients with irritable bowel syndrome (IBS) report lower MMC frequencies and show bacterial overgrowth in the small intestine (Pimentel et al., 2002) implicating alterations in the mucus environment.

## Animal Models of Mucus Impairment

Preclinical models have demonstrated that abnormalities in GI structure and function are associated with altered mucus production. For example, colonic mucus layer thickness is decreased alongside progressive inflammation in a mouse model of colitis (Petersson et al., 2011). In the absence of an inner mucus layer, bacteria can penetrate deep into the epithelial crypts and interact with the colonic epithelium (Johansson et al., 2008) which can exacerbate disease. Furthermore, multiple studies report that alterations in mucus secretory processes result in an underdeveloped colonic inner mucus layer, often associated with sparsely filled goblet cells and an increased susceptibility to colitis (An et al., 2007; Park et al., 2009; Stone et al., 2009; Fu et al., 2011; Tsuru et al., 2013; Bergstrom et al., 2014).

### Muc-2 Knockout Mice

Mice lacking the mucus protein MUC2 (MUC2^−/−^ mice) lack an inner colonic mucus layer despite the presence of goblet cells and other mucus layer components. Interestingly, Rahman and colleagues showed changes in colonic innervation in mice expressing a point mutation in *Muc-2* (Rahman et al., 2015) highlighting interactions between mucus production and innervation of the GI tract. Knockout mice also exhibit altered intestinal cell maturation, migration, and abnormal intestinal crypt morphology (Velcich et al., 2002). These mice develop adenomas and rectal tumors as well as increased infiltration of neutrophils and lymphocytes, loose stools, diarrhea with blood, rectal prolapses, and fail to thrive (Velcich et al., 2002). In the longer term, these mice also show increased susceptibility to developing colon cancer (Velcich et al., 2002; van der Sluis et al., 2006).

### Cystic Fibrosis

Patients with cystic fibrosis are commonly diagnosed with concomitant GI abnormalities including meconium ileus and distal intestinal obstruction syndrome (Colombo et al., 2011) due to an increase in secreted mucus volume, mucus dehydration, and increased viscosity that contributes to blockage of the small intestine. Both mucus buildup and reduced mucus movement occur in these patients due to dysregulated mucus secretion. Cystic fibrosis is caused by mutations in the gene encoding the Cystic Fibrosis Transmembrane conductance Regulator (CFTR) channel important for mucus hydration. These mutations cause defective chloride ion transport out of epithelial cells and dehydration of mucus overlying the epithelium. In patients, mucus remains tightly attached to the small intestinal epithelium and peristaltic movements fail to propel the mucus forward within the GI tract. In keeping with these changes, an increased bacterial load has been observed in cystic fibrosis patients (O'Brien et al., 1993), likely due to the elevated volume and viscosity of mucus that provides an ideal environment for commensal microbes.

Mouse models expressing CFTR mutations also display severe intestinal dysfunction and a mucus layer that is firmly attached to the mucosal epithelium (Grubb and Gabriel, 1997; Seidler et al., 2009; Frizzell and Hanrahan, 2012). Since a prominent role of mucus is to trap and transport bacteria to the distal regions of the gastrointestinal tract via peristalsis, animal models provide an excellent experimental tool to investigate the effects of mucus perturbation on microbial dysbiosis.

### Hirschsprungs Disease

Extreme effects of neuronal loss on goblet cell function and on mucus layer properties have been observed in Hirschsprung disease, a life-threatening developmental disorder where the distal colon lacks enteric neurons due to the failure of neural crest cells to completely migrate during gastrointestinal development. Patients with Hirschsprung disease have a reduced mucin turnover rate, a decreased goblet cell population and reduced expression of *Spdef* and *Krueppel like factor 4* which drive goblet cell differentiation and maturation (Aslam et al., 1997a,b; Nakamura et al., 2018). These findings highlight the importance of the ENS in the development and function of mucus-producing goblet cells in the clinical setting.

Mouse models of Hirschprung Disease additionally provide evidence for neural-mucus interactions. For example, endothelin receptor B knockout mice (Ednrb^−/−^ mice) along with mice expressing a mutation in the RET gene that encodes the receptor for the glial cell line-derived neurotrophic factor (GDNF) are well-characterized models which have been examined for alterations in mucus and goblet cell structure. Mice lacking endothelin receptor B, known for its role in angiogenesis and neurogenesis, show colonic aganglionosis resembling the clinical presentation. Ednrb^−/−^ mice showed an increase in both goblet cell numbers and size as well as increased expression of *Spdef* and Math 1 transcription factors in the distal colon (Thiagarajah et al., 2014). In addition, the absence of *Ednrb* in mice alters mucus structure as evidenced by reduced permeability to 200 nm nanoparticles *in vitro* (Thiagarajah et al., 2014; Yildiz et al., 2015). Furthermore, significant differences in the commensal microbiome were also present in this model (Ward et al., 2012).

The absence of GDNF signaling in mice similarly results in a severely underdeveloped ENS. Furthermore, these mice have altered mucus composition and mucus retention (Porokuokka et al., 2019). Overall, these clinical and animal model data illustrate involvement of the nervous system in the regulation of goblet cell differentiation and maturation as well as influencing mucus properties.

## Neurological Disorders and Mucus Dysfunction

Patients with neurological disorders frequently present with coexistent bowel diseases but whether this is due to nervous system changes *per se* or additional downstream effects such as dysbiosis, immune dysregulation and/or altered mucus production is uncertain. Gut disorders are often associated with, and precede, the core diagnostic symptoms of autism, Parkinson's disease, Alzheimer's disease, and Multiple Sclerosis (Pfeiffer, 2003; Buie et al., 2010; Preziosi et al., 2013; Coggrave et al., 2014). Severe gastrointestinal dysfunction can be debilitating, exacerbate core symptoms of neurological disease, and decrease quality of life. Thus, clarifying the role of the nervous system in mucus production and maintenance could improve understanding of the pathophysiology of neurological disease. Furthermore, modulating mucus properties to optimize probiotics and microbial engineering could provide additional “psychobiotic” therapeutic options for these disorders.

**Figure 3:**
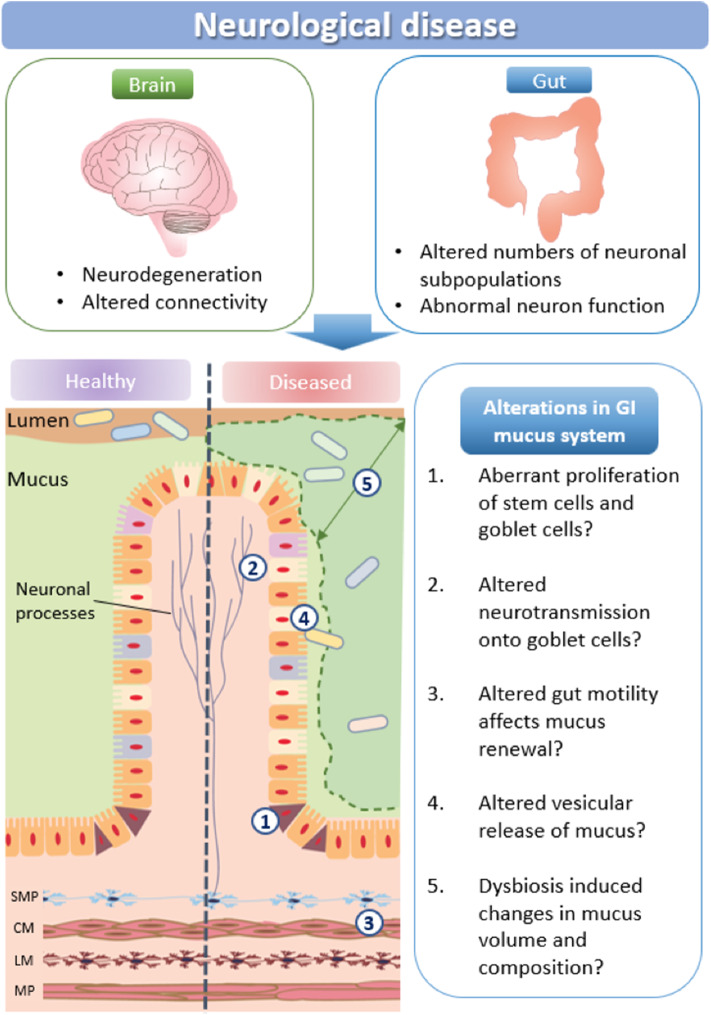
How neurological disease may impact mucus production. Schematic representation of potential changes in mucus production and microbial communities in neurological disorders. SMP, submucosal plexus; CM, circular muscle; MP, myenteric plexus; LM, longitudinal muscle.

A major function of the intestinal mucus layer is to form a barrier between the intestinal epithelium and the luminal content to protect the intestine from pathogenic invasion. A number of biological pathways influence mucus production and volume: (i) stem cell proliferation and subsequent maturation of goblet cells is influenced by the SPDEF transcription factor and the Wnt/notch signaling pathways, as well as neural activity; (ii) multiple neurotransmission pathways directly activate mucus release from goblet cells, including via muscarinic receptors; (iii) motility driven by the enteric nervous system can also affect mucus renewal; (iv) vesicular signaling molecules govern mucus release; and (v) microbes are integral in maintaining mucus homeostasis (Figure 3).

### Developmental Pathways

Key developmental pathways implicated in neurological disease are involved in goblet cell maturation, mucus production and release. For example, the Spdef and Wnt/Notch signaling pathways, known to be crucial for neuronal development in the brain, also influence stem cell maturation in the GI tract. As Spdef regulates the terminal differentiation of goblet cells and Paneth cells (Noah et al., 2010) alterations in these pathways would influence goblet cell turnover and numbers (Lo et al., 2017), therefore modulating mucus properties. The Wnt-beta catenin pathway is also associated with neurological disease (Sani et al., 2012; Zhang et al., 2012, 2014; Ferrari et al., 2014; Huang et al., 2015; Hoseth et al., 2018). This pathway stimulates the synaptic expression and localization of neuroligin-3, a synaptic adhesion protein associated with autism spectrum disorder (Medina et al., 2018). Wnt signaling pathways are also implicated in Parkinson's Disease via interactions with PARK genes (Berwick and Harvey, 2012). Although potential changes in goblet cell number and morphology or mucus properties have not been studied in animal models of autism or several other models of neurological disorders, we predict that Wnt-mediated pathways are altered in the gastrointestinal tract and affect mucus properties, thereby contributing to patient GI symptoms.

### Protein Misfolding

Due to the high levels of protein produced, mucus production processes within goblet cells are susceptible to protein misfolding, retention in the endoplasmic reticulum (ER), and ER stress. Protein misfolding is known to trigger the unfolded protein response (UPR), which is associated with chronic inflammation and autoimmune changes in neurodegenerative diseases such as PD, Alzheimer's disease, and multiple sclerosis (Mhaille et al., 2008; Matus et al., 2011). Accordingly, protein misfolding could result in altered production and apoptosis of goblet cells, therefore affecting mucus properties.

### Vesicle-Associated Proteins

Biological pathways required for neurotransmission and mucus release share molecular components. Multiple neurological disorders are associated with gene mutations that impair neuronal communication via synapses, therefore mutations in the brain potentially affect mucus properties in the gastrointestinal tract. Examples of mucus release components that overlap with synaptic neurotransmitter systems include syntaxin, Munc 18, VAMP, and SNAP proteins. These vesicle-associated proteins are commonly expressed at neuronal synaptic membranes and have been identified as being mutated in neurological disorders (syntaxin; ASD, SNAP; ADHD, Munc 18; epilepsy/ASD (Guerini et al., 2011; Durdiaková et al., 2014; Hamada et al., 2017). Changes in the function of these proteins will not only contribute to brain disorders but may also disrupt vesicular secretion of mucus. Further investigation of mucus properties is therefore warranted in these models and in patients with neurological disorders that potentially express mutations in these and related synaptic genes.

### Mucosa-Associated Microbial Dysbiosis

In neurological disease, changes in mucus properties could additionally alter commensal microbial populations. Dysbiosis has been reported for the mucus-residing microbiome in patients with various neurological disorders including autism, Parkinson's disease, Alzheimer's disease, and multiple sclerosis (Table 2). Because dysbiosis can alter gut barrier function (i.e., via altering mucus thickness), this could contribute to disease progression. Microbial populations influence mucus hydration by releasing enzymes that modify mucus structural networks. Microbes release enzymes that degrade mucus, and this enzymatic cleavage of mucin complexes expands and hydrates the mucus 3-dimensional structure. For example, increased release of mucin-degrading enzymes due to an overgrowth of mucus-residing bacteria (such as *Akkermansia muciniphila*) increases mucus thickness and strengthens the protective mucosal barrier (Ottman et al., 2017). An additional effect of increasing mucus thickness may be reduced nutrient absorption. Such an increase could be beneficial (i.e., in the case of obesity) but detrimental in neurodegenerative diseases such as multiple sclerosis and Parkinson's Disease (Cani, 2018).

**Table 2:** Altered mucosal microbiome in patients with neurological disease.

**Neurological disorder**	**Gastrointestinal dysfunction**	**Altered mucosal microbiome (↓↑ abundance)**		**References**
Autism spectrum disorder	Constipation, diarrhea, functional abdominal pain, food allergies, bloating	↓ Akkermansia muciniphila ↓ Bifidobacteria species		Wang et al., 2011
		↑ Mucosa-associated Clostridiales (Lachnospiraceae and Ruminococcaceae) ↓ Dorea, Blautia, Sutterella		Luna et al., 2017
		↑ Burkholderia ↓ Neisseria		Kushak et al., 2017
		↑ Sutterella		Williams et al., 2012
		↓ Bacteroidetes ↑ Firmicutes (↑*Ruminococcaceae* ↑ *Lachnospiraceae*)		Williams et al., 2011
Parkinson's disease	Constipation	↓ Faecalibacterium (*Blautia, Coprococcus*, and *Roseburia*) ↑ Pro-inflammatory Proteobacteria of the genus *Ralstonia*		Keshavarzian et al., 2015
		↓*Dorea*, ↓ *Bacteroides*, ↓ *Prevotella*, ↓ *Faecalibacterium*, ↓ *Bacteroides massiliensis*, ↓ *Bacteroides coprocola*, ↓ *Stoquefichus* ↓*Blautia glucerasea*, ↓ *Dorea longicatena*, ↓ *Bacteroides dorei*, ↓ *Bacteroides plebeus*, ↓ *Prevotella copri*, ↓ *Coprococcus eutactus*, ↓ *Ruminococcus callidus*	↑*Christensenella*, ↑ *Catabacter*, ↑ *Lactobacillus*, ↑ *Oscillospira*, ↑ *Bifidobacterium*, ↑ *Christensenella minuta*, ↑ *Catabacter hongkongensis*, ↑ *Lactobacillus mucosae*, ↑ *Ruminococcus bromii*, ↑ *Papillibacter cinnamivorans*	Petrov et al., 2017
		↓ Prevotellaceae		Scheperjans et al., 2015
		↑*Akkermansia muciniphila*		Heintz-Buschart et al., 2018
Alzheimer's disease	Constipation, incontinence	↑*Akkermansia muciniphila* ↑*Prevotella denticola*		Zhuang et al., 2018
		↓ Firmicutes and Bifidobacterium ↑ Bacteroidetes		Vogt et al., 2017
		↑ Escherichia/Shigella (pro-inflammatory) ↓ E. rectale (anti-inflammatory)		Cattaneo et al., 2017
Multiple sclerosis	Constipation, diarrhea	↑*Methanobrevibacter* ↑*Akkermansia muciniphila* ↓*Butyricimonas*		Jangi et al., 2016
		↑*Akkermansia muciniphila*,		Berer et al., 2017
		↓ Faecalibacterium		Cantarel et al., 2015
		↑*Akkermansia muciniphila*, ↑ *Acinetobacter calcoaceticus* ↓*Parabacteroides distasonis*		Cekanaviciute et al., 2017

*Arrows indicate an increase or decrease in abundance of bacteria*.

#### Autism

Autism spectrum disorder is a neurodevelopmental disorder characterized by impaired social interactions and restrictive and repetitive behavior. In 2018, 1 in 59 children are diagnosed with autism in the United Status. GI dysfunction is a major comorbidity for autism patients (Kohane et al., 2012; Chaidez et al., 2014; McElhanon et al., 2014) and includes symptoms such as abdominal pain, diarrhea, constipation, and bloating. Altered levels of mucosa-associated bacterial species are reported in autism patients with GI dysfunction with *Akkermansia muciniphila* Dorea, Blautia, *Sutterella Neisseria* having decreased abundance, while mucosa-associated Clostridiales (*Lachnospiraceae* and *Ruminococcaceae*), Burkholderia, *Ruminococcaceae, Lachnospiraceae*, and Sutterella have increased abundance (Wang et al., 2011; Williams et al., 2011, 2012; Kushak et al., 2017; Luna et al., 2017).

#### Parkinson's Disease

Parkinson's disease (PD) is the second most common neurodegenerative disease observed in people over 60 years of age (de Lau and Breteler, 2006). In addition, PD is increasingly correlated with GI disorders prior to the onset of characteristic motor symptoms such as tremor and coordination of complex movement. Although the pathophysiology of PD remains unclear, the accumulation of α-synuclein appears to cause neuronal death (Kirik et al., 2002; Braak et al., 2003). Parkinson's patients with colonic inflammation also showed α-synuclein deposits in their colon (Holmqvist et al., 2014). The mucosal biopsy samples of PD patients showed increased abundance of *Akkermansia muciniphila*, and Ralstonia, and a decrease in abundance of Faecalibacterium (Blautia, Coprococcus, Roseburia) and Prevotella (Keshavarzian et al., 2015; Scheperjans et al., 2015; Petrov et al., 2017; Heintz-Buschart et al., 2018).

#### Alzheimer's Disease

Alzheimer's disease is an increasingly prevalent neurodegenerative disease characterized by progressive cognitive decline and also reported to have comorbid GI dysfunction. Patients with Alzheimer's disease who also had symptoms indicative of IBS showed dysbiosis involving increased abundance of mucolytic bacteria including *Akkermansia muciniphila* and *Prevotella denticola* (Zhuang et al., 2018). Similarly stool samples of Alzheimer patients examined for targeted bacteria showed an increase in the abundance of *Escherichia/Shigella* (pro-inflammatory taxa) and a decrease in abundance of *E. rectale* (anti-inflammatory taxa) (Cattaneo et al., 2017). Microbial dysbiosis in Alzheimer's disease has been implicated in increasing gut permeability, which may influence systemic inflammation and impairment of the blood brain barrier (Vogt et al., 2017; Kowalski and Mulak, 2019).

#### Multiple Sclerosis

Multiple sclerosis involves an aberrant immune system that causes inflammation and results in demyelination in the central nervous system. Multiple studies in patients with multiple sclerosis have found increased abundance of mucosal bacteria including *Akkermansia muciniphila, Methanobrevibacter*, and *Acinetobacter calcoaceticus* and decreased abundance of *Butyricimonas*, Faecalibacterium, and *Parabacteroides distasonis* (Cantarel et al., 2015; Jangi et al., 2016; Berer et al., 2017; Cekanaviciute et al., 2017). Such alterations in the mucosal microbiome potentially favor the growth of pathogenic bacteria that alter the composition of the mucus layer and therefore may exacerbate core symptoms of these disorders (Camara-Lemarroy et al., 2018; Buscarinu et al., 2019)

## Conclusion

In summary, multiple pathways relevant to mucus homeostasis may be impacted by nervous system impairments in neurological disease. Furthermore, altered mucus properties could contribute to the widespread observations of microbial dysbiosis in autism, Parkinson's Disease, Alzheimer's Disease, and multiple sclerosis, and potentially exacerbate core symptoms. Overall, this review highlights that mucus properties could be impaired in neurological disease and provides new avenues for clinically relevant research into GI dysfunction in these disorders.

## Author Contributions

All authors contributed to the design and drafting of the final manuscript.

## Conflict of Interest

The authors declare that the research was conducted in the absence of any commercial or financial relationships that could be construed as a potential conflict of interest.
